# Hypersensitivity to antiretroviral drugs a case report

**DOI:** 10.1186/2045-7022-4-S3-P104

**Published:** 2014-07-18

**Authors:** Maria Sousa, Susana Cadinha, Margarida Mota, Tiago Teixeira, Daniela Malheiro, JP Moreira Silva

**Affiliations:** 1 Centro Hospitalar Vila Nova de Gaia/Espinho, Allergy Department, Portugal; 2 Centro Hospitalar Vila Nova de Gaia/Espinho, Infectious Diseases Department, Portugal

## Background

ARVT (antiretroviral treatment) improved the prognosis of patients with HIV (Human Immunodeficiency Virus) infection. Antiretroviral drugs may be responsible for HR (hypersensitivity reactions) varying in severity, clinical manifestations and frequency.

## Methods

We report a case of a 47-year-old woman, with diagnosis of HIV infection since 2009 (HLA B5701 negative) that started ARVT in 2011. On the 2nd day of treatment with Tenofovir, Emtricitabine and Nevirapine she developed pruritic exanthema and palpebral edema. Two weeks later the Infecciologist discontinued ARVT and prescribed AH (antihistamines) and CS (oral corticosteroids) with symptoms resolution. One month later treatment with Tenofovir and Emtricitabine was restarted in association with Darunavir and Ritonavir. There was a reproducible reaction on the 2nd day of treatment. After the reaction subsided, the patient restarted Darunavir and Ritonavir in association with Abacavir and Lamivudine. On the 2nd day she developed palpebral edema and discontinued ARVT once again. She was then referred to our Drug Allergy Clinic for suspected hypersensitivity to ARVT.

## Results

PT (patch tests) with the suspected drugs were positive for Emtricitabine and Tenofovir and revealed only erythema to Lamivudine. PT performed in 7 controls were negative. Negative DPT (drug provocation test) with Darunavir, Ritonavir and Abacavir were followed by home treatment with no adverse reactions. DPT with Nevirapine was positive (pruritic exanthema and palpebral edema). DPT with an alternative drug, Raltegravir, was negative.

## Conclusion

HR to Darunavir, Ritonavir and Abacavir were excluded, so the patient restarted treatment with these drugs. Since combined therapy with 3 or more drugs is recommended to avoid viral resistance, Raltegravir was introduced to complete therapeutic regimen. HR to Nevirapine was confirmed and HR to Emtricitabine and Tenofovir was considered probable based on positive PT. We admit a possible cross-reactivity with Lamivudine.

